# ELISA-Based Detection System for Protein S K196E Mutation, a Genetic Risk Factor for Venous Thromboembolism

**DOI:** 10.1371/journal.pone.0133196

**Published:** 2015-07-17

**Authors:** Keiko Maruyama, Masashi Akiyama, Koichi Kokame, Akiko Sekiya, Eriko Morishita, Toshiyuki Miyata

**Affiliations:** 1 Department of Molecular Pathogenesis, National Cerebral and Cardiovascular Center, Suita, Japan; 2 Department of Clinical Laboratory Sciences, Graduate School of Medical Science, Kanazawa University, Kanazawa, Japan; IIBB-CSIC-IDIBAPS, SPAIN

## Abstract

Protein S (PS) acts as a cofactor for activated protein C in the plasma anticoagulant system. PS Lys196-to-Glu (K196E) mutation is a genetic risk factor for venous thromboembolism in Japanese individuals. Because of the substantial overlap in PS anticoagulant activity between KK (wild-type) and KE (heterozygous) genotypes, it is difficult to identify PS K196E carriers by measuring PS activity. Here, we generated monoclonal antibodies specific to the PS K196E mutant and developed a simple and reliable method for the identification of PS K196E carriers. We immunized mice with a keyhole limpet hemocyanin-conjugated synthetic peptide with Glu196. The hybridoma cells were screened for the binding ability of the produced antibodies to recombinant mutant EGF-like domains of PS (Ile117–Glu283). We obtained three hybridoma cell lines producing PS K196E mutation-specific antibodies. We established a sandwich enzyme-linked immunosorbent assay (ELISA) system in which the PS K196E mutation-specific monoclonal antibody was used as a detection antibody. We measured human plasma samples by using this system and successfully discriminated 11 individuals with the KE genotype from 122 individuals with the KK genotype. The ELISA system using the PS K196E mutation-specific antibody is a useful tool for the rapid identification of PS K196E carriers, who are at a higher risk for venous thromboembolism.

## Introduction

Protein S (PS) is an anticoagulant protein that acts as a cofactor for activated protein C in the proteolytic inactivation of activated coagulation factors Va and VIIIa and as a cofactor for tissue factor pathway inhibitor to efficiently inhibit factor Xa [1–4]. Thus, the reduced PS anticoagulant activity observed in congenital PS deficiency is a genetic risk for venous thromboembolism (VTE). PS circulates in human plasma at a concentration of approx. 25 μg mL^−1^ (~350 nM). Approximately 60% of PS forms a non-covalent 1:1 stoichiometric complex with C4b-binding protein, which results in loss of cofactor function for activated protein C [1,2].

PS and its structural homologue Gas6 are ligands for TAM receptors (Tyro3, Axl, and Mer) and are involved in various pathological conditions such as inflammation, cancer growth, and autoimmune disease [5]. Protein S is involved in the engulfment of phosphatidylserine-exposed apoptotic cells with Mer-expressing macrophages [6,7]. Mice lacking the PS gene show embryonic lethal coagulopathy and vascular defects [8,9].

VTE is a multifactorial disorder resulting from the interaction of acquired and genetic factors. Regarding the genetic factors, factor V Leiden (c.1601G>A, p.R534Q) and prothrombin G20210A mutations are well-known risk factors for VTE in Caucasians [10]. These two mutations do not exist in East Asian populations [11,12]. We and other researchers identified a missense mutation (c.586A>G, p.K196E) in the PS gene as a genetic risk factor for VTE with odds ratios between 3.74 and 8.56 [13–16].

The frequency of E-allele in the Japanese general population is approx. 0.009 [14,16,17]. PS K196E mutation is likely to be specific for Japanese, because it has not been identified in Chinese, Koreans and Caucasians [17,18]. This mutation is located in the second epidermal growth factor (EGF)-like domain of PS and is also called PS K155E mutation (using a nomenclature system of mature protein) or PS Tokushima mutation [19,20].

Heterozygous carriers for PS K196E mutation showed reduced anticoagulant activity within normal limits of antigen levels, indicating type II deficiency [13,16,19–21]. We reported that the PS anticoagulant activities in individuals with the heterozygous (KE) genotype in a Japanese general population were substantially overlapped with those in individuals with the wild-type (KK) genotype; the mean difference of PS anticoagulant activity was only 16% [22]. This finding suggests that PS anticoagulant activity is not a useful marker for the PS K196E mutation.

PS K196E carriers have been identified thus far by genetic analyses such as direct sequencing, genotyping (e.g., TaqMan), and restriction fragment length polymorphism analysis [19–22]. These analyses are accurate but expensive and time-consuming. In addition, they are not routinely available at clinical laboratories. A simple and rapid detection method for PS K196E carriers in the clinical setting remained to be established. In the present study, we developed a sandwich enzyme-linked immunosorbent assay (ELISA) system for detecting a PS K196E mutant in plasma, using a novel monoclonal antibody.

## Materials and Methods

### Ethics Statement

This study was approved by the Institutional Review Boards of the National Cerebral and Cardiovascular Center and the Kanazawa University Graduate School of Medical Science. Written informed consent was obtained from all individuals involved in the study.

### Generation of PS K196E mutation-specific antibodies

Three GANP transgenic mice [23] were immunized with a keyhole limpet hemocyanin-conjugated synthetic 11-amino acid peptide (C^186^KNGFVMLSNE ^196^, mutation underlined) with the K196E mutation. GANP mice express a high level of germinal center-associated nuclear protein and an increased frequency of somatic mutations in the Ig-variable region, and they are thus suitable for high-affinity antibody production [23].

Hybridoma cells were generated by a standard cell fusion technique and plated in 96-well plates by limiting dilution. Immunization and generation of hybridoma cells were performed by TransGenic Inc. (Kumamoto, Japan).

### Expression and purification of recombinant PS proteins

Wild-type and K196E mutant forms of human full-length PS (Ala41–Ser676) (FL-PS-K and FL-PS-E, respectively) and those of four EGF-like domains of PS (Ile117–Glu283) (EGF-PS-K and EGF-PS-E, respectively) were expressed in HEK293S GnTI^−^ cells obtained from the American Type Culture Collection (Manassas, VA) by our use of the mammalian expression vector with a mouse *Nid-1* signal sequence and a C-terminal His tag [24]. For the expression of FL-PS, vitamin K1 (10 mg L^−1^) was added to the culture medium.

Recombinant PS proteins were purified from the culture supernatants by Ni-affinity chromatography. After dialysis in phosphate-buffered saline (PBS, pH 7.4), the proteins were concentrated and used for the binding assay of monoclonal antibodies, the Western blot analysis, and validation of the PS K196E ELISA system. The concentrations of the recombinant proteins were determined from the absorbance at 280 nm assuming an *E*_280_^1%^ = 10.0.

### Screening of PS K196E-specific antibodies

The ELISA plates were coated with 50 μL of 1 μg mL^−1^ EGF-PS-K or EGF-PS-E for 1 h at room temperature (RT). The plates were washed three times with PBS with 0.05% Tween 20 and blocked with 200 μL of 0.5% gelatin in PBS overnight at 4°C. The plates were then incubated with 50 μL of culture supernatants of hybridoma cells for 1 h at RT. After being washed, the plates were incubated with 50 μL of 1 μg mL^−1^ horseradish peroxidase (HRP)-labeled anti-mouse IgG antibody (Kirkegaard & Perry Laboratories, Gaithersburg, MD) for 1 h. After the plates were washed again, 0.5 mg mL^−1^ *o*-phenylenediamine was added to the plates, and absorbance was measured at 490 nm against a reference wavelength of 650 nm.

### Antibody purification and labeling

Three hybridoma cell lines were cultured in RPMI 1640 medium containing 10% fetal bovine serum. The monoclonal antibodies were purified from the culture supernatants using an Ex-Pure Protein G kit (Kyoto Monotech, Kyoto, Japan). Purified antibodies were labeled with HRP using the Peroxidase Labeling Kit NH_2_ (Dojindo Laboratories, Kumamoto, Japan). The concentrations of the monoclonal antibodies were determined from the absorbance at 280 nm assuming an *E*_280_^1%^ = 10.0.

### Binding assay of monoclonal antibodies

For the antibody binding assay, the plates were coated with 50 μL of 0.05 μg mL^−1^ EGF-PS-K or EGF-PS-E or 50 μL of 1.0 μg mL^−1^ FL-PS-K or FL-PS-E for 1 h at RT. After blocking, the plates were incubated with 50 μL of 1.0 μg mL^−1^ monoclonal antibody for 1 h at RT. After the plates were washed, 50 μL of HRP-labeled anti-mouse IgG antibody was added and the plates were incubated for 1 h. After the plates were washed again, 100 μL of 3,3’,5,5’-tetramethylbenzidine (TMB) substrate solution (Kirkegaard & Perry Laboratories) was added. After a 10-min incubation, 100 μL of 1 N HCl was added and the absorbance was measured at 450 nm against a reference wavelength of 650 nm.

### Western blot analysis

The recombinant PS proteins with a C-terminal His tag were subjected to sodium dodecyl sulfate-polyacrylamide gel electrophoresis (SDS-PAGE) (10%–20% gradient gel) and transferred to a polyvinylidene fluoride membrane (Bio-Rad, Hercules, CA). After blocking with 5% skim milk in PBS, the membrane was incubated with 1.0 μg mL^−1^of monoclonal mouse anti-His tag antibody (Cat. No. D291-3, MBL, Nagoya, Japan) or the PS K196E mutation-specific monoclonal antibody 15C8 for 1 h, washed with PBS, and incubated with 0.1 μg mL^−1^ HRP-labeled anti-mouse IgG (Cat. No. 474–1802, Kirkegaard & Perry Laboratories). The chemiluminescence signal was detected by using Immobilon Western Chemiluminescent HRP Substrate (Millipore, Billerica, MA) and an LAS-3000 imager (Fujifilm, Tokyo).

### Plasma samples

We collected DNA and plasma samples from patients with VTE and normal individuals to evaluate the hereditary or acquired risks for the development of VTE, at the Kanazawa University Graduate School of Medical Science. Although DNA samples were obtained from all of the participants of the study, plasma samples were obtained from only 133 individuals consisting of 11 with the mutant KE genotype and 122 with the wild-type KK genotype. Of the 11 individuals with the KE genotype, three had a history of adverse pregnancy outcome, two had a history of a thrombotic event, one was a rheumatoid arthritis patient, and the remaining five were healthy. Of the two individuals with a history of thrombotic events, one suffered from pulmonary embolism and warfarin was administered. The other developed thrombophlebitis in pregnancy, and heparin was administered. None of the individuals with the KK genotype had a history of thrombotic events.

Blood samples were drawn from an antecubital vein through a needle into disposable, siliconized, evacuated plastic tubes containing a 1:10 volume of 3.2% (wt/vol) trisodium citrate. The plasma fractions were separated by centrifugation at 1,710 × *g* for 10 min, and genomic DNA was isolated from peripheral blood leukocytes. The plasma samples were stored at −80°C until measurement.

### Genotyping

The K196E mutation was genotyped using a polymerase chain reaction-restriction fragment length polymorphism (PCR-FFLP) analysis. The mutagenic primers 5′-AAT GGT TTT GTT ATG CTT TCA CAT-3′ (mutation underlined) and 5′- TGT TAG TAT AAG CAC TTA CAT ATC-3′ were employed. Two fragments (27 bp and 128 bp) of the Hsp 92II (Promega, Madison, WI)-digested PCR product from the mutant allele were separated by 8% Synergel (Diversified Biotech, Dedham, MA) electrophoresis.

### The PS K196E ELISA system for the detection of a PS K196E mutant in plasma

The plates were coated with 100 μL of 10 μg mL^−1^ polyclonal rabbit anti-human PS antibody (Cat. No. A0384, Dako, Glostrup, Denmark) in 50 mM carbonate bicarbonate buffer (pH 9.6) and left overnight at 4°C. The plates were washed with Tris-buffered saline (TBS, pH 7.4) with 0.1% Tween 20 and blocked with 200 μL of 1% bovine serum albumin in TBS for 1 h at RT. After blocking, the plates were incubated with TBS alone or with 100 μL of patients’ plasma samples diluted 20-fold using TBS for 1–2 h at RT.

After the plates were washed, the plates were then incubated with 100 μL of 5 μg mL^−1^ HRP-labeled PS K196E mutation-specific monoclonal antibody 15C8 for 1 h. After the plates were washed, 100 μL of TMB substrate solution was added to the plates. After a 10-min incubation, 100 μL of 1 N HCl was added and the absorbance was measured at 450 nm against a reference wavelength of 650 nm. The assay result of each sample was obtained by subtracting the absorbance value of TBS alone from that of each sample.

### Validation of PS K196E ELISA system

We tested our newly developed ELISA system by determining the intra-assay variability, inter-assay variability, dilution linearity, and spiking recovery. Intra- and inter-assay variations were determined as the coefficients of variation (CV). The CV was determined by dividing the standard deviation by the mean of absorbance at 450 nm against a wavelength of 650 nm for each plasma sample. The intra-assay CV was determined by measuring the absorbance of three KE plasma samples five times in a single run. The inter-assay CV was determined by measuring the absorbance of three KE plasma samples in five separate runs.

To examine the linearity of dilution, we serially diluted FL-PS-E from 0 to 2.5 μg ml^−1^ in TBS alone, or a 20-fold diluted PS-deficient plasma sample contained in the STA Staclot Protein S reagent kit (Roche Diagnostics, Mannheim, Germany), or 20-fold diluted KK plasma. In the analytical recovery experiments, we assayed two different 20-fold diluted KE plasma samples in duplicate after the addition of three different amounts of FL-PS-E (25, 50, 100 ng). Serial twofold dilutions from 0 to 2.5 μg mL^−1^ of FL-PS-E in 20-fold diluted KK plasma were used for a standard curve. The recovery was calculated using the formula: (detected concentration/expected concentration) × 100.

## Results and Discussion

We screened supernatants of hybridoma cells from 1,672 wells by preferential binding to a recombinant mutant PS EGF-like domain, EGF-PS-E, compared to its wild-type counterpart, EGF-PS-K. We obtained three monoclonal antibodies, 4B1, 15C8 and 16E3, all of which bound to mutant EGF-PS-E but not to wild-type EGF-PS-K.

**Fig 1:**
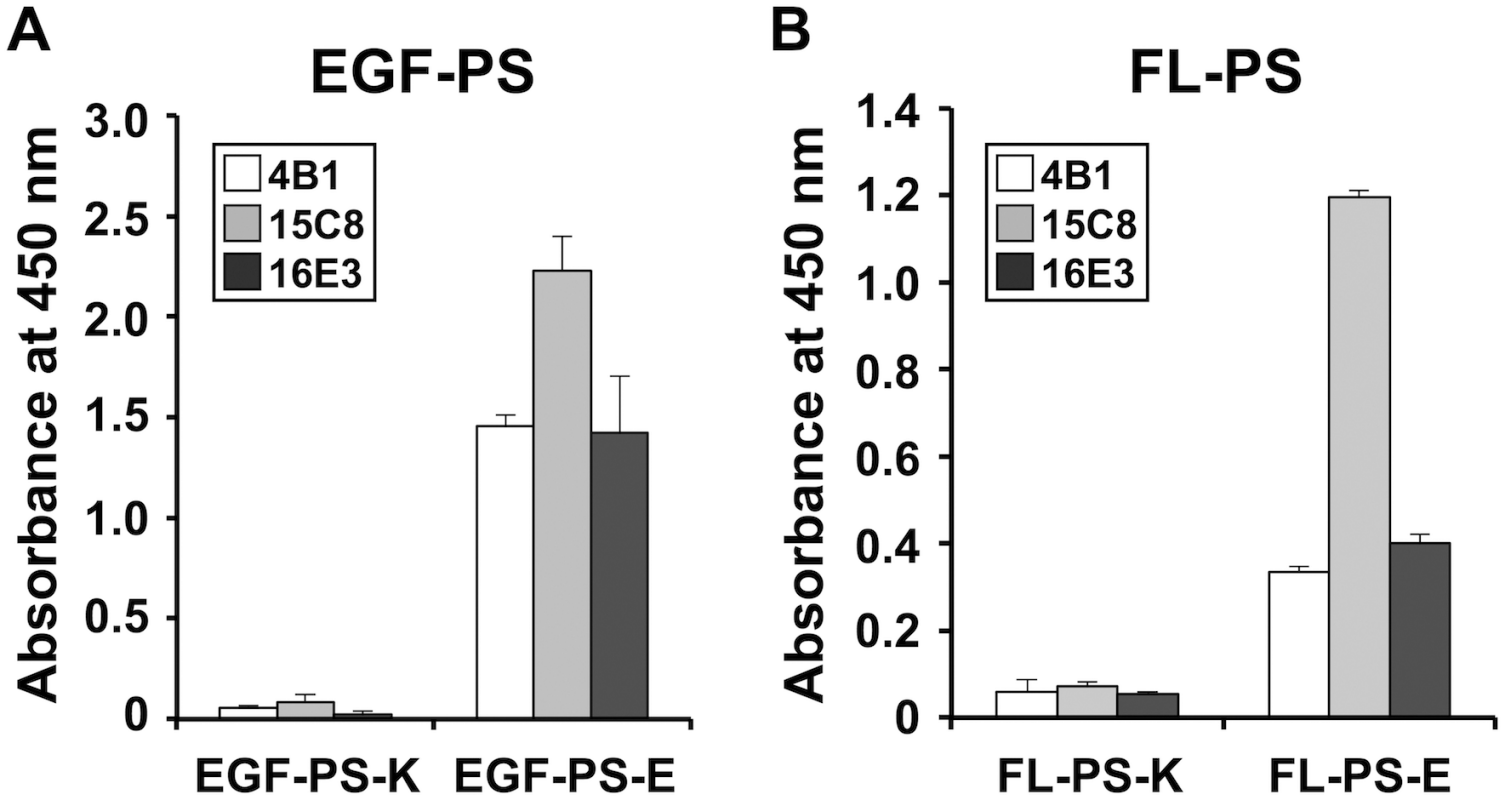
Binding of PS K196E mutation-specific monoclonal antibodies to recombinant PS proteins. The 96-well plates were coated with EGF-PS-K or EGF-PS-E (A) or FL-PS-K or FL-PS-E (B). The plates were then incubated with each of three monoclonal antibodies, 4B1, 15C8, and 16E3. HRP-labeled anti-mouse IgG antibody was used for the second antibody. Bound HRP was developed with TMB substrate, and the absorbance was measured at 450 nm. Error bars: SD (n = 3).

**Fig 2:**
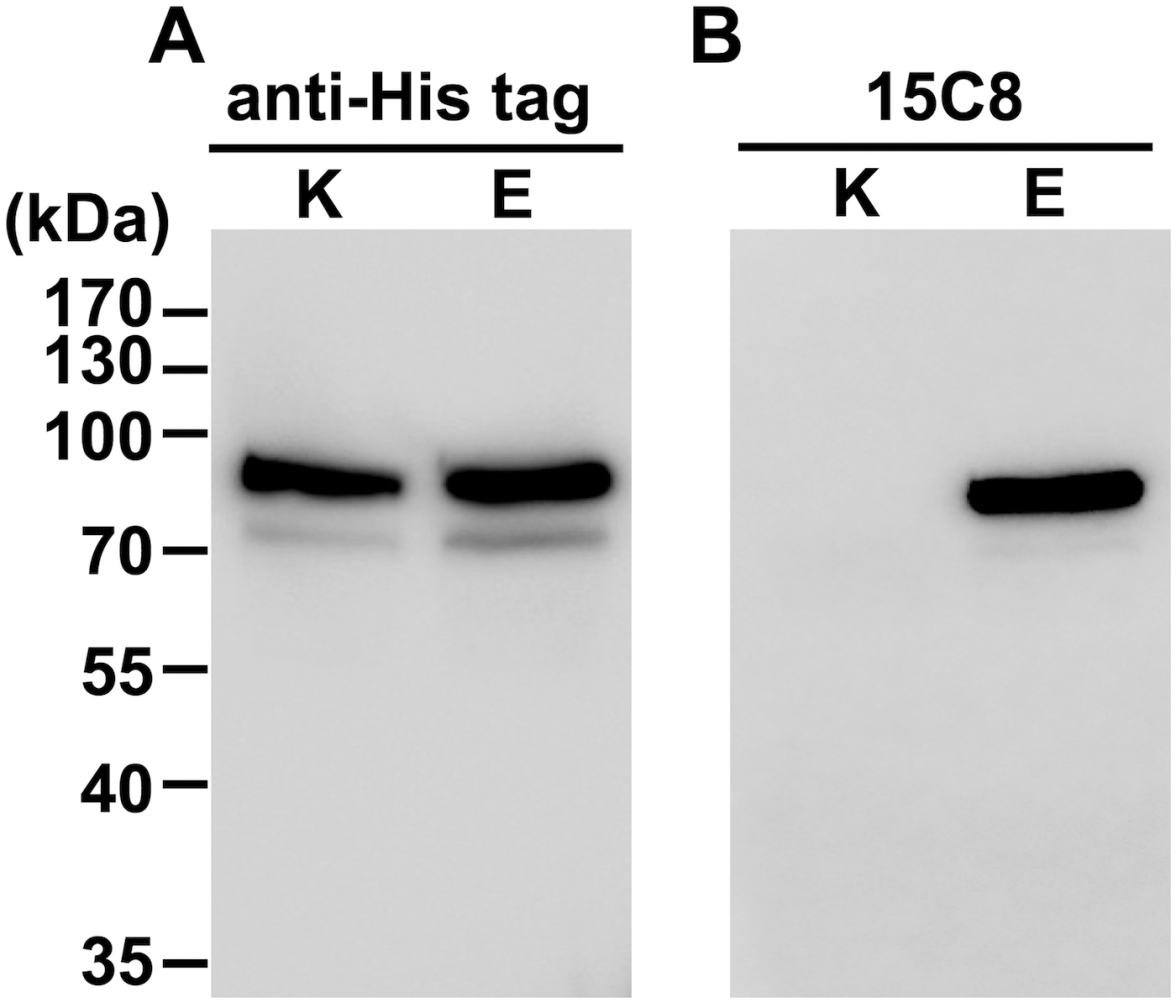
Western blot analysis of the recombinant PS proteins using the PS K196E mutation-specific monoclonal antibody. FL-PS-K and FL-PS-E were separated by SDS-PAGE under reducing conditions, transferred to a membrane, and incubated with the anti-His tag antibody (A) or the PS K196E mutation-specific monoclonal antibody 15C8 (B), and then with HRP-labeled anti-mouse IgG. The proteins were visualized using a chemiluminescent substrate. The molecular weights are shown on the left.

We compared the binding of the three monoclonal antibodies to wild-type and mutant forms of PS EGF domains and full-length PS. All three antibodies bound to the mutant EGF domain EGF-PS-E (Fig 1A) as well as the full-length mutant FL-PS-E (Fig 1B). Among the antibodies, 15C8 bound most strongly to mutant FL-PS-E (Fig 1B) and was thus used for the subsequent experiments. The Western blot analysis showed that an anti-His tag antibody detected both His-tagged wild-type FL-PS-K and mutant FL-PS-E (Fig 2A), whereas the 15C8 antibody specifically detected FL-PS-E (Fig 2B).

**Fig 3:**
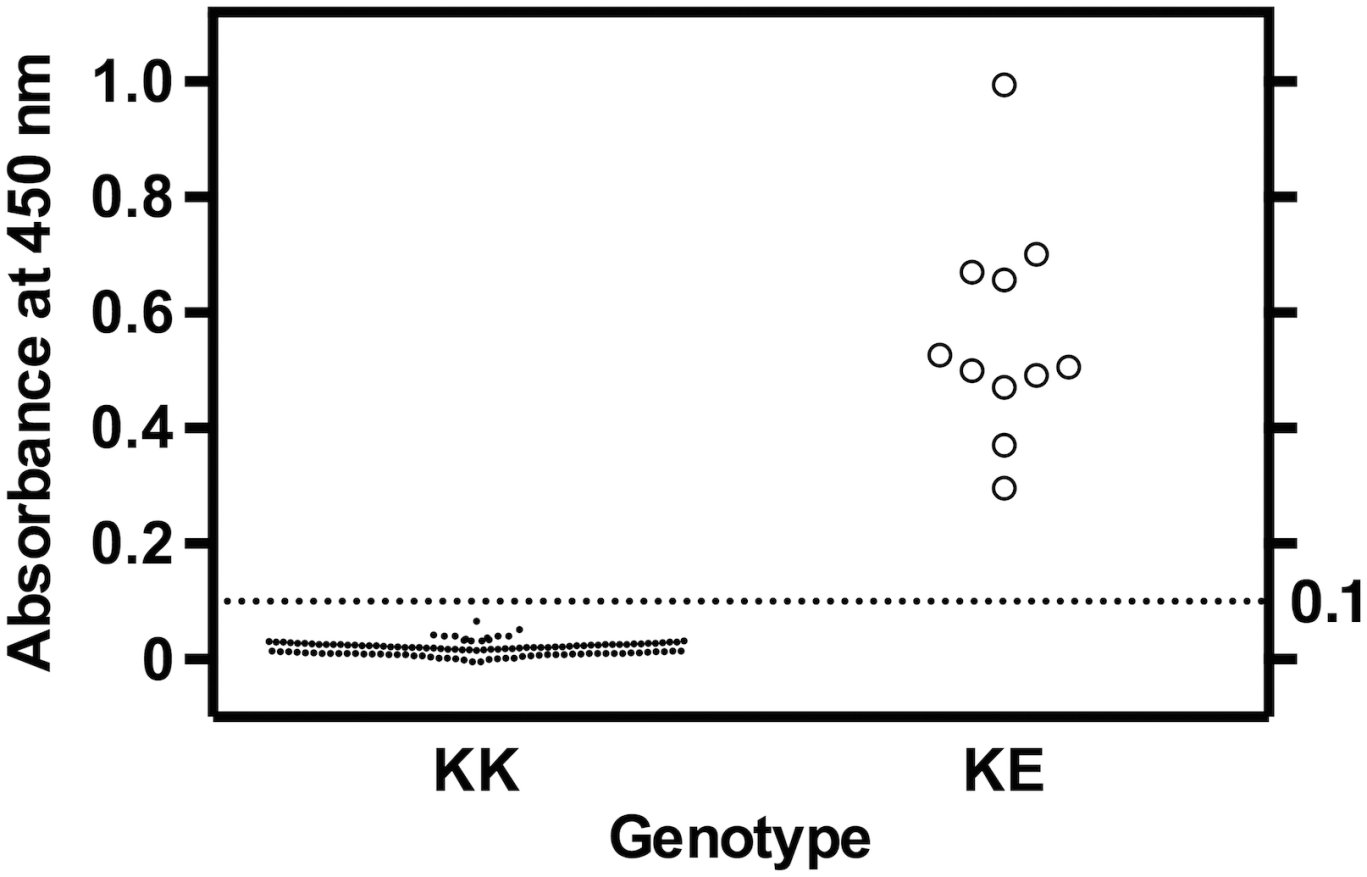
Detection of PS K196E mutant in plasma by the PS K196E ELISA system. Plasma samples diluted 20-fold using TBS were measured by the PS K196E ELISA system. The absorbance ranges of 122 KK and 11 KE plasma samples were −0.01–0.07 and 0.30–1.00, respectively. All data points represent the mean of three wells performed in triplicate.

For widespread clinical laboratory use to identify PS K196E carriers, we developed a sandwich ELISA system for detecting the PS K196E mutant in 20-fold diluted plasma using a PS polyclonal antibody as the capture antibody and the PS K196E mutation-specific monoclonal antibody 15C8 as the detection antibody. In the 133 human plasma samples examined in the present study, all 11 PS K196E heterozygote (KE) samples showed distinctly higher absorbance compared to the 122 wild-type (KK) samples (absorbance range: KK, −0.01–0.07; KE, 0.30–1.00) (Fig 3). In addition, the KE plasma samples obtained from a warfarin-treated individual and a pregnant individual also showed higher absorbance compared to the wild-type samples (absorbance: warfarin, 0.30; pregnancy, 0.37).

The intra-assay CVs and inter-assay CVs obtained from three plasma samples ranged from 1.4% to 3.1% and from 8.1% to 14.7%, respectively. All were within a generally acceptable range (*<*10% for the intra-assay CV and *<*15% for the inter-assay CV) [25, 26]. We therefore concluded that the PS K196E ELISA system can discriminate KE samples from KK samples.

**Fig 4:**
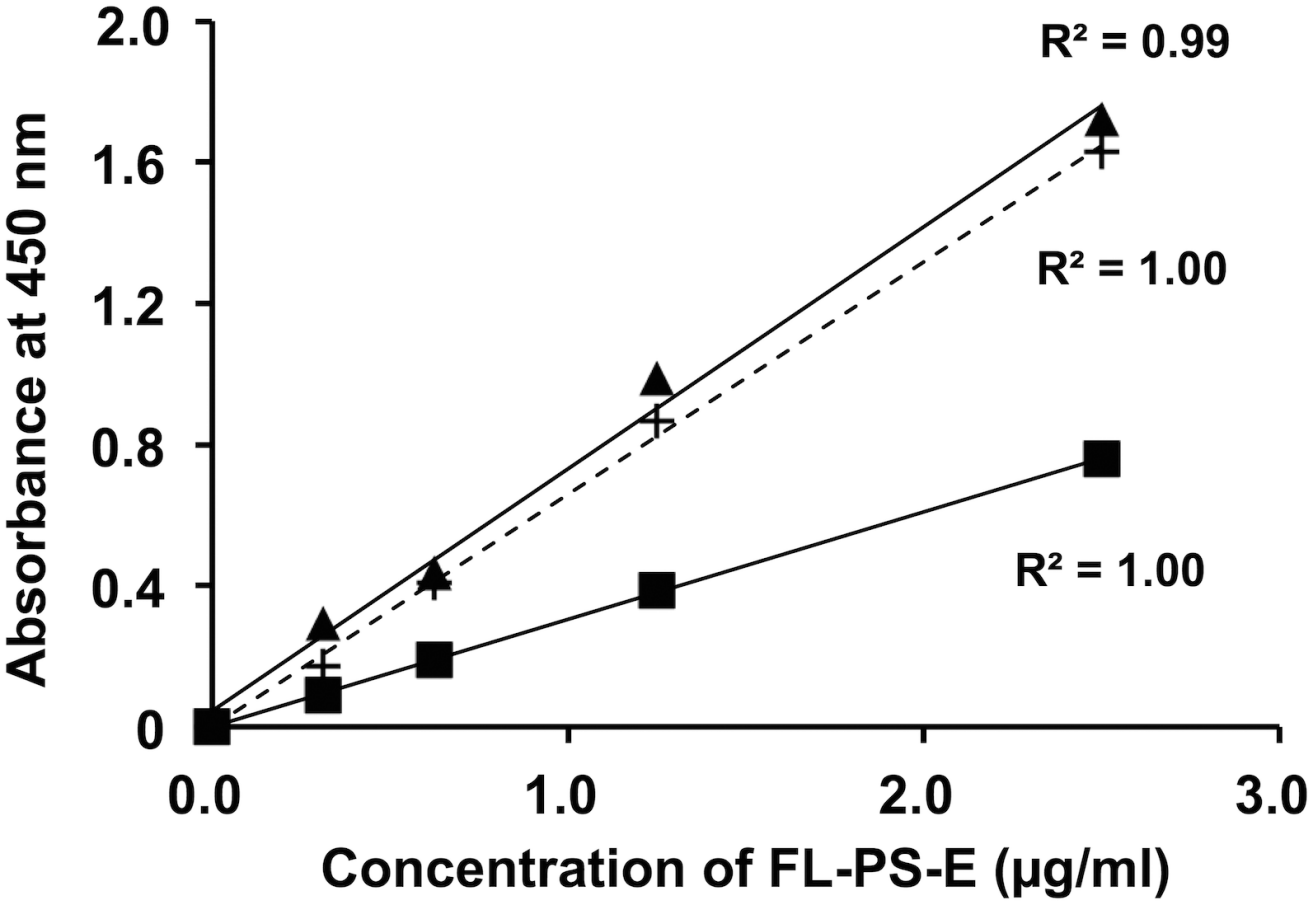
Dilution plots of FL-PS-E in the PS K196E ELISA system. FL-PS-E was serially diluted from 0 μg mL^−1^ to 2.5 μg mL^−1^ in TBS (▲), 20-fold diluted PS-deficient plasma (+), or a 20-fold diluted KK plasma sample (■).

To examine the linearity of the PS K196E concentration in the ELISA system, we made three different standard curves obtained from a series of the FL-PS-E dilution in TBS alone, 20-fold diluted PS-deficient plasma, and 20-fold diluted KK plasma. All gave good linearity (Fig 4, R^2^ = 0.99–1.00). The standard curves prepared from dilution with TBS alone and with the PS-deficient plasma were similar (Fig 4). The FL-PS-E standards diluted with wild-type KK plasma showed lower absorbance values than those with TBS alone or with PS-deficient plasma (Fig 4). Wild-type PS-K and mutant PS-E may compete for binding to a PS polyclonal antibody immobilized on the microplate wells, resulting in the low absorbance values in the FL-PS-E standards diluted with wild-type KK plasma.

We next performed recovery experiments. The recoveries of three different amounts of FL-PS-E (25, 50, and 100 ng) in two different KE plasma samples ranged from 95% to 106% (recovery of FL-PS-E: 25 ng, 95%: 50 ng, 100%: 100 ng, 106%) when the standard curve obtained from the FL-PS-E standards diluted with KK plasma was used. When the standard curves were obtained from the FL-PS-E standards diluted with PS-deficient solution such as TBS or PS-deficient plasma, the recovery was low (22%–76%, 25%–67%, respectively).

The competition between wild-type PS-K and mutant PS-E for binding to the immobilized PS polyclonal antibody raises the limitation of the ELISA system in terms of quantification. That is, the concentration of PS-K in each KE plasma sample will affect the absorbance value in the ELISA system. Currently, we cannot overcome this problem.

Ethnic heterogeneity of genetic mutations in the human genome has been reported, and it is important to consider the ethnic variability of genes for properly assessing the risk of thrombosis and VTE in specific populations [27]. For example, carrier testing for factor V Leiden mutation is now one of the most frequently ordered molecular genetic tests in Caucasian populations [28]. However, this testing is not useful for East Asian populations because these populations do not have the factor V Leiden mutation [11,12]. On the other hand, PS K196E is a genetic mutation found in about 1.8% of the Japanese population [14,16,17], and the prevalence of the mutation in Japanese VTE patients is 5%–10% [13,21,22]. Given the frequency of the PS K196E mutation and its association with VTE, screening for PS K196E carriers will be valuable, especially for high-risk VTE populations such as patients with cardiovascular diseases or cancer. In addition to the clinical application, our PS K196E mutation-specific ELISA system would also be useful in research seeking to identify the association of this mutation with thrombosis-related diseases.

PS type II deficiency is characterized by a reduced anticoagulant activity within normal limits of antigen levels, and it could be expected to show decreased PS-specific activity that can be obtained from the anticoagulant activity divided by the antigen level. An assay system for measuring PS-specific activity was previously developed for identifying the PS K196E mutation [29]. In that study, low PS-specific activity of < 0.69 was observed in individuals with the PS K196E mutation. Unfortunately, this method also identified individuals with low PS-specific activity who had the PS C247F mutation and warfarin-treated patients [29]. Thus, an assay system for PS-specific activity is not specific for identifying individuals with the PS K196E mutation. Our newly developed ELISA system specifically detected the PS K196E mutant and thus will be useful for the identification of PS K196E carriers in clinical settings.

A potential limitation of our mutation-specific ELISA system is that it cannot distinguish the homozygotes from the heterozygotes. Plasma PS antigen levels are expected to be strongly correlated with the absorbance values in the PS K196E ELISA system in homozygotes and heterozygotes with differing slopes. However, the present measurements using 11 KE plasma samples did not show a good correlation between the total PS antigen levels and the absorbance values in the PS K196E ELISA system (data not shown). The reasons for this might be the limited sample size and the heterogeneity of the patients’ background. We should examine the correlation using a much larger sample size in the near future. The discrimination of homozygotes from heterozygotes cannot be achieved using the PS K196E ELISA system at this time.

### Conclusions

We developed a novel ELISA system for detecting a PS K196E mutant in plasma. This system will be a useful tool for identifying PS K196E carriers who are at high risk for VTE in the clinical environment.
